# Survival strategies of cancer cells: the role of macropinocytosis in nutrient acquisition, metabolic reprogramming, and therapeutic targeting

**DOI:** 10.1080/15548627.2025.2452149

**Published:** 2025-01-16

**Authors:** Guoshuai Xu, Qinghong Zhang, Renjia Cheng, Jun Qu, Wenqiang Li

**Affiliations:** a Department of General Surgery, Aerospace Center Hospital, Beijing, China; b Emergency Department, Shengjing Hospital of China Medical University, Shenyang, Liaoning, China; c Department of Intensive Care Medicine, The General Hospital of the Northern Theater Command of the People’s Liberation Army of China, Shenyang, Liaoning, China

**Keywords:** Anti-cancer therapies, macropinocytosis, metabolic reprogramming, methuosis, MTORC1, MTORC2

## Abstract

Macropinocytosis is a nonselective form of endocytosis that allows cancer cells to largely take up the extracellular fluid and its contents, including nutrients, growth factors, etc. We first elaborate meticulously on the process of macropinocytosis. Only by thoroughly understanding this entire process can we devise targeted strategies against it. We then focus on the central role of the MTOR (mechanistic target of rapamycin kinase) complex 1 (MTORC1) in regulating macropinocytosis, highlighting its significance as a key signaling hub where various pathways converge to control nutrient uptake and metabolic processes. The article covers a comprehensive analysis of the literature on the molecular mechanisms governing macropinocytosis, including the initiation, maturation, and recycling of macropinosomes, with an emphasis on how these processes are hijacked by cancer cells to sustain their growth. Key discussions include the potential therapeutic strategies targeting macropinocytosis, such as enhancing drug delivery via this pathway, inhibiting macropinocytosis to starve cancer cells, blocking the degradation and recycling of macropinosomes, and inducing methuosis – a form of cell death triggered by excessive macropinocytosis. Targeting macropinocytosis represents a novel and innovative approach that could significantly advance the treatment of cancers that rely on this pathway for survival. Through continuous research and innovation, we look forward to developing more effective and safer anti-cancer therapies that will bring new hope to patients.

**Abbreviation**: AMPK: AMP-activated protein kinase; ASOs: antisense oligonucleotides; CAD: carbamoyl-phosphate synthetase 2, aspartate transcarbamylase, and dihydroorotase; DC: dendritic cell; EGF: epidermal growth factor; EGFR: epidermal growth factor receptor; ERBB2: erb-b2 receptor tyrosine kinase 2; ESCRT: endosomal sorting complex required for transport; GAP: GTPase-activating protein; GEF: guanine nucleotide exchange factor; GRB2: growth factor receptor bound protein 2; LPP: lipopolyplex; MTOR: mechanistic target of rapamycin kinase; MTORC1: mechanistic target of rapamycin kinase complex 1; MTORC2: mechanistic target of rapamycin kinase complex 2; NSCLC: non-small cell lung cancer; PADC: pancreatic ductal adenocarcinoma; PDPK1: 3-phosphoinositide dependent protein kinase 1; PI3K: phosphoinositide 3-kinase; PIK3C3: phosphatidylinositol 3-kinase catalytic subunit type 3; PtdIns(3,4,5)P_3_: phosphatidylinositol-(3,4,5)-trisphosphate; PtdIns(4,5)P_2_: phosphatidylinositol-(4,5)-bisphosphate; PTT: photothermal therapies; RAC1: Rac family small GTPase 1; RPS6: ribosomal protein S6; RPS6KB1: ribosomal protein S6 kinase B1; RTKs: receptor tyrosine kinases; SREBF: sterol regulatory element binding transcription factor; TFEB: transcription factor EB; TNBC: triple-negative breast cancer; TSC2: TSC complex subunit 2; ULK1: unc-51 like autophagy activating kinase 1; UPS: ubiquitin-proteasome system.

## Introduction

Endocytosis is a fundamental cellular process by which cells internalize extracellular substances [1,2]. It encompasses several distinct mechanisms, including clathrin-mediated endocytosis [3], lipid raft-mediated endocytosis [4] and macropinocytosis [5], etc, each of which plays unique roles in cellular homeostasis. Clathrin-mediated endocytosis is highly selective and involves the formation of coated pits that internalize specific ligands and receptors [3]. Lipid raft-mediated endocytosis is independent of clathrin but involves caveolin and associated cytoskeletal proteins to form clathrin-independent vesicles [4]. This pathway is highly specific to certain receptors due to the molecular composition of lipid rafts. However, macropinocytosis is a form of nonselective endocytosis where the cell engulfs large volumes of extracellular fluid and dissolved solutes by forming membrane ruffles that enclose the fluid, creating large vesicles called macropinosomes [5–7]. In cancer cells, macropinocytosis stands out due to its nonselective nature and ability to engulf large volumes of extracellular fluid. Studies have demonstrated that cells with high macropinocytic activity can internalize an amount of extracellular fluid equivalent to their own volume within just 1 to 2 h [8]. This allows cancer cells to efficiently acquire the nutrients required to sustain their rapid proliferation and aggressive metabolism. Unlike other endocytic pathways, macropinocytosis can accommodate a broad range of macromolecules, such as proteins and growth factors, making it an essential process for nutrient scavenging in nutrient-poor tumor microenvironments [9–11]. Under normal physiological conditions, free amino acids and glucose in the extracellular environment are typically the preferred sources of nutrients for cells. However, when an organism experiences starvation and nutrient levels in tissues drop below the cellular requirements, cells may activate macropinocytosis as a mechanism to adapt to this nutrient-deficient state [12–14]. Shao and colleagues found that placental trophoblast cells activate macropinocytosis during maternal starvation, capturing and degrading serum proteins to meet the nutritional needs of the fetus [15]. Cancer cells have a much faster metabolism and proliferation rate compared to normal cells, thus requiring a large amount of energy and materials. The glucose and amino acids available in the extracellular environment are often insufficient to meet their demands. Therefore, many cancer cells utilize macropinocytosis as a supplementary source of nutrients, participating in energy metabolism [13,16–19]. Given that cancer cells exhibit high levels of macropinocytic activity, which is typically low in normal cells except for immune cells, selectively inhibiting macropinocytosis in cancer cells could potentially suppress their growth and proliferation, thereby reducing their energy metabolism. This strategy is what is referred to as “starving cancer cells to death”.

**Figure 1.**
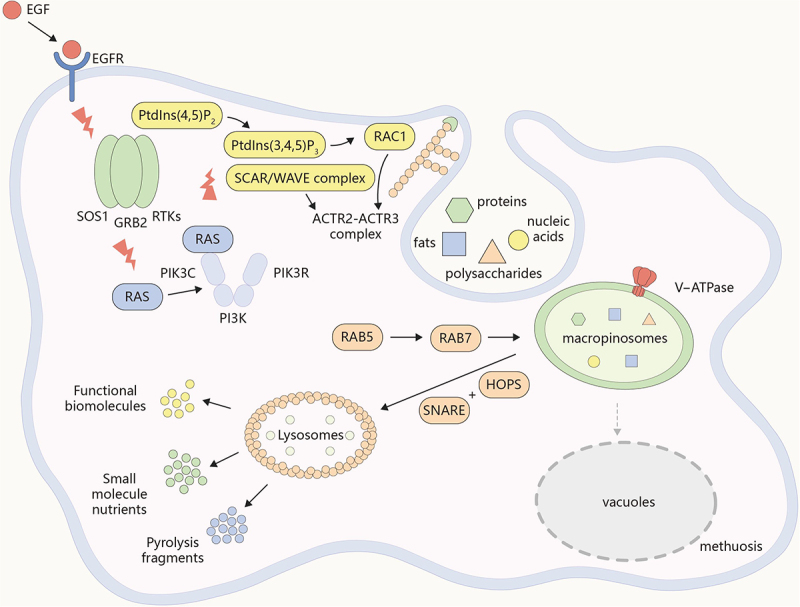
The process of macropinocytosis. Including signal activation (such as via EGF), actin cytoskeleton remodeling, formation of macropinosomes, maturation of macropinosomes, degradation and recycling of macropinosomes, and macropinosomes undergoing homotypic fusion to form larger vacuoles (methuosis).

This review delves into the endocytic mechanism of macropinocytosis (Figure 1) and analyzes its functions and regulatory mechanisms within cancer cells, especially focusing on the central role of the MTORC1 and MTORC2 signaling pathway in this process. The high activity of macropinocytosis in certain cancer cells provides them with a unique survival strategy, enabling them to acquire essential nutrients in the nutrient-deprived tumor microenvironment [13,20–23]. This dependence on macropinocytosis not only reveals a potential weakness in cancer cell metabolism, but also provides new perspectives for the development of innovative anti-cancer therapeutic strategies.

## The process of macropinocytosis

### The initiating process of macropinocytosis

#### Signal activation

Signal activation is the initial step in macropinocytosis where extracellular signals (such as growth factors, cytokines, or other molecules) bind to cell surface receptors, initiating a cascade of intracellular events that lead to macropinocytosis. The process of macropinocytosis is often initiated by the activation of receptor tyrosine kinases (RTKs) upon the binding of extracellular ligands. One of the most well-studied ligands in this context is EGF (epidermal growth factor). When EGF binds to its receptor (EGFR), it induces receptor dimerization, bringing the intracellular kinase domains into close proximity. This proximity allows for the cross-phosphorylation or autophosphorylation of specific tyrosine residues within the cytoplasmic domain of the receptors. These phosphorylated tyrosine residues serve as docking sites for downstream signaling molecules, effectively triggering the signaling cascade necessary for macropinocytosis [24–26]. Commisso and colleagues highlighted how growth factor receptor engagement, particularly EGFR, initiates RAS and phosphoinositide 3-kinase (PI3K)-AKT signaling pathways to promote actin cytoskeleton rearrangement and membrane ruffling, crucial steps in the formation of macropinosomes [27].

Following autophosphorylation, RTKs recruit receptor proteins that propagate the signal downstream. One of the critical receptor proteins involved in this process is GRB2 (growth factor receptor bound protein 2), which binds to the phosphorylated tyrosine residues on RTKs [28]. GRB2, in turn, is constitutively associated with SOS1 (SOS Ras/Rac guanine nucleotide exchange factor 1), a guanine nucleotide exchange factor (GEF) [28,29]. The RTK-GRB2-SOS1 complex facilitates the activation of RAS, a small GTPase, by promoting the exchange of GDP for GTP on RAS [30] (Figure 1). Studies have shown that activated RAS (GTP-bound RAS) then serves as a pivotal signaling node that activates multiple downstream pathways [30,31].

One of the critical pathways activated by RAS is the PI3K pathway. PI3K, a heterodimer consisting of a regulatory subunit (PIK3R) and a catalytic subunit (PIK3C), is recruited to the plasma membrane upon activation [32]. RAS interacts with the catalytic subunit of PI3K, leading to its activation [33]. PI3K catalyzes the phosphorylation of phosphatidylinositol-(4,5)-bisphosphate (PtdIns(4,5)P_2_) to produce phosphatidylinositol-(3,4,5)-trisphosphate (PtdIns(3,4,5)P_3_), a lipid second messenger that plays a crucial role in membrane signaling [30,34] (Figure 1).

PtdIns(3,4,5)P_3_ serves as a docking site for proteins with pleckstrin homology domains, including GEFs such as TIAM1 (T-lymphoma invasion and metastasis 1) and PREX1 (PtdIns-3,4,5-trisphosphate dependent Rac exchange factor 1) [35]. These GEFs facilitate the activation of RAC1 (Rac family small GTPase 1), another small GTPase, by promoting the exchange of GDP for GTP on RAC1 [35,36]. Activated RAC1 (GTP-bound RAC1) initiates a series of downstream signaling events essential for actin cytoskeleton remodeling, which is critical for the formation of membrane ruffles. The signal activation process in macropinocytosis is a finely tuned sequence of molecular events that begins with ligand binding to RTKs. This process involves the coordinated action of multiple signaling molecules and pathways, including the RAS, PI3K, and RAC1 pathways.

### Actin cytoskeleton remodeling

Activated RAC1 translocates to the plasma membrane. Once there, it engages in intricate interactions with various downstream effectors, amongst which stands out the SCAR/WAVE complex, which acts as a key partner in this intricate signaling cascade. The SCAR/WAVE complex consists of several subunits, including WASF1 (WASP family member 1), ABI2 (abl interactor 2), NCKAP1 (NCK associated protein 1), CYFIP2/PIR121 (cytoplasmic FMR1 interacting protein 2), and BRK1/HSPC300 (BRICK1 subunit of SCAR/WAVE actin nucleating complex) [37]. These subunits form a stable complex that is crucial for its regulatory functions. Upon binding to active RAC1, the SCAR/WAVE complex undergoes a conformational change that releases its autoinhibited state [37–39]. Ding and colleagues found that RAC1 binding is stabilized by an improved strategy and used cryo-EM to determine the structure of the three different states of SCAR/WAVE complex binding to RAC1 [40]. This change is critical for exposing the verprolin homology, cofilin homology, acidic (VCA) domain of WAVE, which is essential for ACTR2/Arp2-ACTR3/Arp3 activation.

The ACTR2-ACTR3 complex is composed of seven subunits: two actin-related proteins (ACTR2 and ACTR3) and five additional subunits (ARPC1 to ARPC5) [41]. The VCA domain of WASF1 interacts directly with the ACTR2-ACTR3 complex. The verprolin/V domain binds to actin monomers, the cofilin/C domain binds to the ACTR2-ACTR3 complex, and the acidic/A domain facilitates the activation of the complex [42]. The binding of the VCA domain to the ACTR2-ACTR3 complex induces a conformational change that enhances its actin-nucleating activity [41,42]. Koestler et al. found that this activation is further stabilized by the binding of actin monomers to the ACTR2-ACTR3 complex [43]. The ACTR2-ACTR3 complex binds to the sides of preexisting actin filaments and nucleates new filaments at a 70-degree angle, forming a dendritic (branched) network [44]. The newly formed branches are stabilized by additional actin-binding proteins, ensuring the integrity and functionality of the branched actin network [42,44–46]. This branching is critical for creating the force needed to push the plasma membrane outward during processes such as macropinocytosis.

Actin cytoskeleton remodeling is a critical step in macropinocytosis, enabling the formation of membrane ruffles and the subsequent uptake of extracellular fluid and particles. This process involves a well-coordinated interplay of signaling molecules and actin-binding proteins, orchestrated by the activation of RAC1, the SCAR/WAVE complex, and the ACTR2-ACTR3 complex (Figure 1).

### Formation of macropinosomes

Membrane ruffling is a critical step in macropinocytosis, characterized by the formation of dynamic, wave-like protrusions on the cell surface. These ruffles capture extracellular fluid and particles, leading to the formation of macropinosomes. The polymerization of these actin filaments generates the force necessary to push the plasma membrane outward, forming broad, sheet-like protrusions known as lamellipodia or membrane ruffles [26,29,47]. Actin-binding proteins play a crucial role in the regulation of actin cytoskeleton dynamics and macrocytosomal formation. They control the polymerization, depolymerization, and organization of actin filaments, enabling the precise formation of structures such as membrane ruffles during macropinocytosis (Table 1) [48–55]. The membrane ruffles are highly dynamic, constantly extending and retracting. As membrane ruffles extend and fold back toward the cell surface, they form cup-shaped structures that encapsulate extracellular fluid and particles [56]. These cup-shaped structures eventually close and pinch off from the plasma membrane, forming large vesicles known as macropinosomes [8,42,47]. Following the formation of macropinosomes, the actin filaments depolymerize, allowing the recycling of actin monomers for future rounds of polymerization and membrane ruffling.

**Table 1.** Actin-binding proteins.

CFL (cofilin)[49,57]	General Characteristics: Regulation of Activity: Function: Role in Macropinocytosis:	CFL (cofilin) is a small, actin-binding protein, typically around 19 kDa, composed of approximately 150 amino acids. The activity of CFL is regulated by phosphorylation. Activated RAC1, through PAK1 (p21 (RAC1) activated kinase 1), leads to the activation of LIMK (LIM domain kinase). When phosphorylated on serine 3 by LIMK, CFL is inactive and cannot bind to actin. CFL binds to actin filaments (F-actin) and increases the rate of actin filament turnover by promoting actin depolymerization. By meticulously severing actin filaments, CFL generates new barbed ends that serve as optimal sites for further actin polymerization
PFN (profilin) [51,58]	General Characteristics: Regulation of Activity: Function: Role in Macropinocytosis:	PFN (profilin) is a small, globular protein, approximately 15 kDa in size, consisting of about 125 amino acids. PFN regulation in macropinocytosis involves the PI3K-AKT-PFN pathway. PFN binds to actin monomers (G-actin) and facilitates their addition to the growing ends of actin filaments (F-actin), promoting actin polymerization. PFN also acts as a nucleotide exchange factor, accelerating the exchange of ADP for ATP on G-actin, thus replenishing the pool of ATP-actin monomers available for polymerization. By increasing the local concentration of ATP-actin monomers, PFN promotes the rapid polymerization of actin filaments required for the extension of membrane ruffles.
FMN (formin) [59]	General Characteristics: Regulation of Activity: Function: Role in Macropinocytosis:	FMN (formin) proteins are large, multi-domain proteins typically composed of 1,000 to 1,500 amino acids. FMNs are activated by Rho family GTPases. Binding of these GTPases to the GTPase-binding domain (GBD) induces a conformational change, allowing the FMN homology 2 (FH2) domain to nucleate and elongate actin filaments. FMNs are a family of actin-nucleating proteins that promote the nucleation and elongation of unbranched actin filaments. They function by binding to the barbed end of actin filaments and facilitating the addition of actin monomers. FMNs stabilize the newly nucleated actin filaments and promote their elongation, contributing to the formation and maintenance of the actin network necessary for membrane ruffling.
Capping Proteins [55]	General Characteristics: Regulation of Activity: Function: Role in Macropinocytosis:	Capping proteins are typically heterodimeric proteins composed of two subunits, known as alpha and beta subunits. Each subunit is approximately 30–36 kDa in size. Capping Protein regulation in macropinocytosis involves the PI3K-SGK1-capping proteins pathway. Capping proteins bind to the barbed ends of actin filaments, preventing the addition or loss of actin monomers. Capping proteins ensure that the actin filaments involved in membrane ruffling are of optimal length and stability, providing the structural support needed for the formation of macropinosomes.

### Maturation of macropinosomes

Macropinosomes are large, fluid-filled vesicles formed during macropinocytosis, a process by which cells internalize extracellular fluid and solutes [58]. Following their formation, macropinosomes undergo a complex maturation process involving a series of biochemical and structural changes. The newly formed macropinosomes are large, irregularly shaped vesicles that contain extracellular fluid, solutes, and any particles present in the extracellular environment [60–62]. Early macropinosomes retain many surface proteins and lipids from the plasma membrane, including signaling receptors and phosphoinositides such as PtdIns(4,5)P_2_ [60,61]. The Buckley group reported on the role of the WASH complex in macropinocytosis and phagocytosis, particularly its involvement in the early stages of surface receptor recycling from macropinosomes [63]. These vesicles are highly dynamic, continuously undergoing changes in size, shape, and composition as they mature.

One of the first steps in macropinosome maturation is the acidification of the vesicle lumen. Proton pumps, specifically vacuolar-type H^+^-translocating ATPases (V-ATPases), are recruited to the macropinosome membrane [64]. The activity of V-ATPases lowers the internal pH of the macropinosome from near-neutral (pH 7) to more acidic levels (pH 5–6) [65,66]. The acidic environment activates a range of hydrolytic enzymes necessary for the degradation of internalized macromolecules. Acidification also prepares macropinosomes for fusion with other intracellular vesicles, such as endosomes and lysosomes, facilitating further maturation. The Perera team reported the intricate transcriptional control governing the macroautophagy/autophagy-lysosome system in pancreatic cancer metabolism. They highlighted the indispensable nature of an acidic milieu for the activation of hydrolytic enzymes, such as glycosidases and phosphatases, which play a fundamental role in the degradation of macromolecular substances within the cancer cell’s metabolic machinery [67]. Early macropinosomes can fuse with early endosomes, which provide additional membrane components and enzymatic machinery. This fusion is mediated by RAB5. RAB5 is a small GTPase associated with the early endosomal membranes, promoting fusion with early endosomes and recruiting effectors necessary for early maturation [68]. As maturation progresses, macropinosomes transition to late endosomes. This transition involves RAB5 being replaced by RAB7. RAB7 is another small GTPase that regulates the fusion of late endosomes with lysosomes [69]. Yap et al. found that RAB7 facilitates the fusion of macropinosomes with lysosomes, leading to the formation of mature, degradative compartments [70].

The maturation of macropinosomes is a highly regulated, multi-step process that involves acidification, membrane trafficking, and fusion with lysosomes. These processes ensure the proper degradation and recycling of internalized material, playing essential roles in nutrient sensing, immune responses, and cellular homeostasis (Figure 1).

### Degradation and recycling of macropinosomes

Mature macropinosomes eventually fuse with lysosomes, forming hybrid organelles where the internalized content is degraded by lysosomal enzymes. This fusion is mediated by soluble N-ethylmaleimide-sensitive factor attachment protein receptor/SNARE proteins and the homotypic fusion and protein sorting/HOPS complex, which facilitate membrane tethering and fusion [71,72]. Lysosomes contain a variety of hydrolytic enzymes, including proteases, lipases, and nucleases, which are delivered to macropinosomes upon fusion. The lysosomal enzymes degrade proteins, lipids, nucleic acids, and other macromolecules within the macropinosome [73]. These basic building blocks are then available for cellular metabolism and biosynthetic processes. The sorting of internalized cargo is a complex process involving various sorting machineries, such as the endosomal sorting complex required for transport (ESCRT) complex [74]. Bienvenu et al. found that ESCRT complex helps sort ubiquitinated cargo for degradation or recycling [74]. During sorting, some internalized membrane proteins are sequestered into intraluminal vesicles within multivesicular bodies. These vesicles can either fuse with lysosomes for degradation or with the plasma membrane for the release of exosomes [20]. The degradation products are transported out of the lysosome and into the cytoplasm through specific transporters located on the lysosomal membrane, where they can be utilized for various cellular functions, including biosynthesis and energy production [75]. Examples of such transporters include SLC38A9 (solute carrier family 38 member 9) for amino acids [76] and NPC1 (NPC intracellular cholesterol transporter 1) for cholesterol [77]. Similarly, specific membrane proteins and receptors are sorted and recycled back to the plasma membrane [78,79]. The recycling process involves the formation of recycling endosomes, which bud off from the macropinosome or endolysosome and transport the receptors back to the cell surface [2,80]. The internalized membrane components of macropinosomes are recycled, contributing to membrane turnover and remodeling. The recycling of membrane proteins and lipids allows the cell to adapt its surface composition in response to environmental changes, facilitating processes such as cell migration, adhesion, and signal transduction.

The maturation of macropinosomes is followed by a complex and highly regulated decomposition and recycling process. This process involves lysosomal fusion and degradation, the release and transport of nutrients, cargo sorting and receptor recycling (Figure 1). These mechanisms ensure the efficient utilization of internalized materials, maintain cellular homeostasis, and facilitate cellular responses to environmental changes. Understanding these processes provides valuable insights into the functional roles of macropinosomes in cellular physiology and pathology.

### Abnormal accumulation and fusion of macropinosomes—dysregulated macropinocytosis

Macropinosomes do not recycle or effectively fuse with lysosomes, leading to their excessive accumulation and fusion into large vacuoles [81–83]. This is a specific mode of cell death, called Methuosis [81–83]. Methuosis is usually induced by the excessive activation of macropinocytosis within cells. When this endocytosis loses control, it may cause methuosis. Macropinosomes undergo homotypic fusion to form larger vacuoles [81]. Dysregulation of ion channels and transport proteins on the vacuole membrane can lead to increased permeability and osmotic imbalance, promoting vacuole expansion [81,83]. Additionally, lysosomal dysfunction or the fusion with lysosomes can prevent the degradation of vacuole contents, leading to further vacuole enlargement [83]. Ultimately, these vacuoles occupy most of the cell’s volume. The accumulation of large vacuoles can sequester essential nutrients and organelles, leading to metabolic stress and energy depletion [84]. The large vacuoles can displace cytoplasmic organelles and disrupt the cell’s structure and function [85]. The physical expansion of the large vacuoles exerts mechanical stress on the cytoskeleton and plasma membrane, resulting in cell rupture [85–87]. The physiological functions of the cell are severely affected, leading to the collapse of cell structure and function, and ultimately resulting in cell death [86]. Nara and colleagues found that hyperstimulation of the macropinocytosis can lead to increased fluid uptake. Instead of shrinking, the vesicles swell, creating large vacuoles and catastrophic cellular swelling that leads to plasma membrane rupture, cell lysis, and death [88]. Unlike apoptosis, methuosis does not involve the activation of caspases and DNA fragmentation [83].

Although methuosis as a cell death mechanism has been recognized, its precise molecular mechanisms and regulatory pathways still require further study. The discovery of methuosis provides a new perspective for understanding the diversity and complexity of cell death and may offer opportunities for developing new therapeutic approaches. However, research in this field is still evolving, and many questions remain unanswered.

#### Levels of macropinocytosis in different cancer cell types

Macropinocytosis plays a significant role in cellular nutrient acquisition, particularly in cancer cells [17]. Notably, the dependency on macropinocytosis varies considerably across different types of cancer. By examining the variability in macropinocytosis levels, we gain insight into how different cancers utilize this process to adapt metabolically and survive in challenging environments. Here’s a closer look at several specific cancers and how macropinocytosis functions within each.

Research shows that *RAS*-mutant cancers often exhibit elevated levels of macropinocytosis compared to other cancers [27]. In these cells, macropinocytosis is not only a nutrient acquisition mechanism but also a critical survival strategy under nutrient-deprived and hypoxic conditions typical of the tumor microenvironment. Approximately 90% of pancreatic ductal adenocarcinoma (PDAC) cases harbor mutations in the *KRAS* gene, leading to constitutive activation of pathways that promote macropinocytosis [89]. Through macropinocytosis, PDAC cells take up extracellular proteins, such as albumin, which are broken down into amino acids, including glutamine, a crucial nutrient in glucose-scarce conditions. Kamphorst’s research has shown that PDAC cells deprived of glutamine become increasingly dependent on albumin uptake through macropinocytosis, effectively utilizing this pathway to fuel rapid cell proliferation [14]. Inhibiting macropinocytosis in PDAC models has demonstrated a marked reduction in tumor growth, supporting its potential as a therapeutic target [90].

The level of macropinocytosis in non-small cell lung cancer (NSCLC) is variable and often subtype-dependent. *KRAS*-mutant adenocarcinomas show higher macropinocytosis levels than squamous cell carcinomas, reflecting differences in metabolic demands and adaptation mechanisms [91]. This variation suggests that while macropinocytosis may contribute to NSCLC growth, it is not universally essential across all lung cancer cells. Studies have shown that combining macropinocytosis inhibitors with conventional therapies may enhance the effectiveness of NSCLC treatment, particularly in resistant subtypes [92].

Glioblastoma, a highly aggressive brain tumor, exhibits moderate levels of macropinocytosis. It is characterized by rapid growth and resilience in low-nutrient, hypoxic environments [93]. In glioblastoma, macropinocytosis supports cell survival in the nutrient-poor and hypoxic tumor environment. Studies suggest that the ability of glioblastoma cells to modulate their macropinocytotic activity in response to environmental stress contributes to the tumor’s notorious resistance to treatment [94].

While breast cancer generally displays low macropinocytosis levels, triple-negative breast cancer (TNBC) is an exception. TNBC cells exhibit moderate levels of macropinocytosis, particularly under nutrient-deprived conditions, as a compensatory mechanism to import proteins for nutrient recycling [95]. This adaptive response is especially evident in hypoxic tumor regions, where conventional metabolic pathways are insufficient to support growth [96]. Although TNBC does not rely on macropinocytosis to the same degree as PDAC or NSCLC, the pathway still offers a potential therapeutic target for this difficult-to-treat cancer [97].

Prostate cancer has low basal levels of macropinocytosis, reflecting its lower metabolic demands compared to more aggressive cancers like PDAC and NSCLC [32]. However, under specific stress conditions, such as androgen deprivation therapy/ADT, prostate cancer cells may upregulate macropinocytosis to adapt metabolically [98]. Consequently, this pathway is not a primary nutrient acquisition mechanism for prostate cancer cells but serves as a secondary support system under therapeutic stress.

The variability in macropinocytosis levels across cancer types is governed by several molecular mechanisms. Mutations in genes like *KRAS* and alterations in the PI3K-AKT-MTOR (mechanistic target of rapamycin kinase) pathway play significant roles in modulating macropinocytosis. Additionally, cancer cells in nutrient-poor environments often upregulate macropinocytosis as an adaptive mechanism, highlighting the complex interplay between genetic mutations and environmental pressures in determining macropinocytosis activity. In the next part we will detail the regulatory mechanisms mediating macropinocytosis.

#### Interactions between macropinocytosis and MTOR in nutrient metabolism in cancer cell

**Figure 2.**
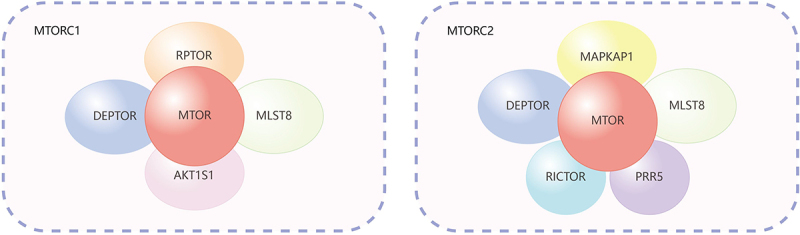
Composition of MTORC1 and MTORC2 core components. MTORC1: MTOR: a serine/threonine kinase that is the catalytic core of the complex. RPTOR: essential for substrate specificity and recruiting downstream effectors. MLST8: stabilizes the kinase domain of MTOR. AKT1S1/PRAS40: a negative regulator that inhibits MTORC1 activity when not phosphorylated. DEPTOR: another negative regulator that inhibits MTOR activity. MTORC2: MTORC2 also shares the MTOR kinase, MLST8, and DEPTOR, but contains unique components RICTOR, PRR5/PROTOR, and MAPKAP1/MSIN1. RICTOR: a defining component of MTORC2. PRR5/PROTOR1-PRR5L/PROTOR2: accessory components of MTORC2. MAPKAP1/MSIN1: essential for MTORC2 integrity and function. It interacts with RICTOR and contributes to the complex’s ability to phosphorylate specific substrates.

Cancer cells divide rapidly, requiring substantial amounts of energy and biosynthetic precursors. Tumors often outgrow their blood supply, leading to hypoxia (low oxygen levels) and nutrient deprivation, particularly in the tumor core [96]. To survive and proliferate, cancer cells have developed a variety of metabolic adaptations, one of which is the enhanced uptake of extracellular nutrients via macropinocytosis. This process is closely linked to the MTOR signaling pathway, which is central to regulating cell growth, metabolism, and survival. The structural details of MTORC1 and MTORC2 can be found in Figure 2. MTOR complex 1 (MTORC1) serves as a master regulator of cellular metabolism, ensuring that cells adapt their metabolic activities in response to fluctuating environmental conditions. In cancer cells, the activation of MTORC1 is particularly dependent on nutrients obtained through macropinocytosis, which is vital for meeting the high metabolic demands of rapidly proliferating tumors. Although MTOR complex 2 (MTORC2) also contributes to cellular regulation, its role is often considered complementary to the more dominant functions of MTORC1 in controlling metabolic processes. Consequently, this section will explore the interplay between macropinocytosis and MTOR in regulating nutrient metabolism in cancer cells, especially under nutrient-poor conditions. The discussion will primarily focus on the function and regulation of MTORC1, highlighting its central role in controlling cellular metabolism, while also addressing the secondary contributions of MTORC2 in this context.

### Upstream regulation of MTORC1

#### Growth factor/insulin stimulation – PI3K-AKT pathway activated by growth factors/insulin

**Figure 3.**
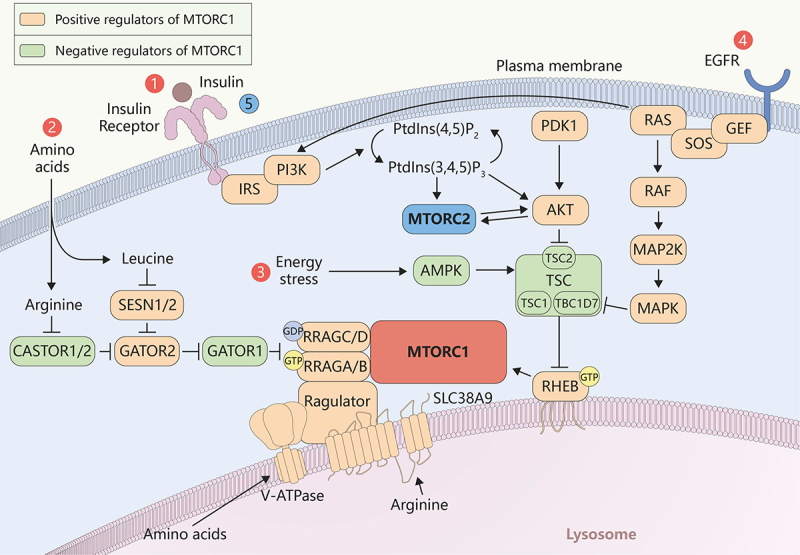
Upstream regulation of MTORC1 and MTORC2. PI3K-AKT pathway activated by insulin; amino acids activate MTORC1 via the RRAG GTP enzyme; energy stress activated AMPK pathway; RAS activated RAF-MAP2K-MAPK pathway; MTORC2 as an effector of insulin-PI3K signaling.

Growth factors such as insulin and EGF activate RTKs, leading to PI3K activation. Activated RTKs recruit and activate PI3K, which converts PtdIns(4,5)P_2_ to PtdIns(3,4,5)P_3_ in the plasma membrane [24]. PtdIns(3,4,5)P_3_ serves as a docking site for proteins with pleckstrin homology (PH) domains, including AKT and PDPK1/PDK1 (3-phosphoinositide dependent protein kinase 1) is a protein kinase that, when recruited to the cell membrane by PtdIns(3,4,5)P_3_, can further activate AKT [35]. PDPK1 phosphorylates AKT on the Thr308 residue in the activation loop, partially activating it. Full activation of AKT requires phosphorylation at the Ser473 residue by MTORC2 [99,100]. Once activated, AKT is transferred to the lysosomal surface. Subsequently, active AKT on the lysosomal membrane phosphorylates and inhibits TSC2/tuberin (TSC complex subunit 2), a GTPase-activating protein (GAP) for RHEB in a larger complex comprised of TSC1, TSC2 and TBC1D7 [101–103]. Under basal conditions, the TSC complex is localized to the lysosomal membrane, where it inhibits RHEB activity, thus inhibiting MTORC1 [100,103]. While phosphorylation of TSC2 by AKT disrupts the TSC complex, impairing its ability to localize to the lysosome. Manning et al. first identified TSC2 as a target of the PI3K-AKT pathway [104]. The phosphorylated TSC2 dissociates from the lysosomal membrane, leading to the disassembly or relocalization of the TSC complex within the cytoplasm. With the TSC complex dissociated from the lysosome, RHEB remains in its GTP-bound active state. Active RHEB directly interacts with MTORC1 and activates it, which in turn regulates nutrient acquisition, substance synthesis, cell growth, and energy metabolism [103,105,106] (Figure 3).

#### Nutrient sensing—amino acids activate MTORC1 via the RRAG GTP enzyme

In cancer cells, the activation of MTORC1 is tightly linked to nutrient availability, particularly amino acids, which can be supplied through macropinocytosis. Amino acids, particularly leucine and arginine, are sensed by the RRAG GTPases on the lysosomal membrane, leading to the activation of MTORC1 [107]. SLC38A9, a lysosomal transmembrane protein, interacts with the V-ATPase and activates the Ragulator by sensing lumenal arginine [76,108]. Interestingly, Castellano et al. found that SLC38A9, a lysosomal transmembrane protein, is required for cholesterol-mediated MTORC1 activation and functions independently of its arginine-sensing capability [77]. SESN1 (sestrin1)/SESN2 (sestrin2) sense leucine [109], and CASTOR1/CASTOR2 sense arginine [110] in the cytosol. When leucine levels are low, SESN1/SESN2 are active and inhibit the GATOR2 complex. Upon binding leucine, SESN1/SESN2 undergo a conformational change that releases their inhibition of GATOR2. Valenstein and colleagues reported that the three-dimensional structure of the human GATOR2 complex determined using electron cryomicroscopy [111]. The GATOR2 complex consists of five proteins, including MIOS, WDR24, WDR59, SEH1L, and SEC13 [111]. GATOR2 acts as a positive regulator of MTORC1 by inhibiting the GATOR1 complex. In the presence of leucine and arginine, SESN1/SESN2 and CASTOR1/CASTOR2, respectively, are inactivated, which allows GATOR2 to inhibit GATOR1. Jiang et al. found that the Ring domains are crucial for the integrity of the GATOR2 complex and the activation of MTORC1, and that the Ring domain of WDR24 can prevent its own ubiquitination [112]. Leucine stimulation can disrupt the binding of SESN2 to the Ring domain of WDR24, promoting the ubiquitination of NPRL2, a process essential for GATOR2-mediated GATOR1 inactivation. The study also found that the loss or deletion of the WDR24 Ring domain can prevent MTORC1 activation, leading to defects and death during mouse embryonic development [112]. The GATOR1 complex consists of DEPDC5, NPRL2, and NPRL3 [113]. GATOR1 acts as a GAP for RRAGA/RRAGB, converting them from the GTP-bound active state to the GDP-bound inactive state, thereby inhibiting MTORC1 [114]. When GATOR1 is inhibited by GATOR2, RRAGA/RRAGB remain in their GTP-bound state, while RRAGC/RRAGD are in their GDP-bound state. The active RRAG GTPase heterodimer binds to the Ragulator complex, positioning MTORC1 for activation. The Ragulator complex consists of LAMTOR1 to LAMTOR5 proteins and acts as a scaffold that anchors RRAG GTPases to the lysosomal membrane. The active RRAG GTPase heterodimer recruits MTORC1 to the lysosomal membrane [115,116]. MTORC1 is translocated to the lysosomal surface, where it encounters its activator, RHEB. Active RHEB-GTP binds directly to MTORC1, leading to its activation [117] (Figure 3). Yang et al. discovered a distinct mechanism in their study [118]. They found that RHEB binds at a site distant from the MTOR kinase active site, but induces global conformational changes. Through an allosteric effect, this binding rearranges the active site residues, thereby accelerating catalysis. Additionally, the study provided new insights into the substrate recruitment mechanism of MTORC1 [118].

#### Energy status—activation of AMPK

AMP-activated protein kinase (AMPK) is a crucial energy sensor in cells that maintains energy homeostasis [119–121]. Under conditions of low energy (high AMP:ATP ratio), AMPK is activated and promotes catabolic processes to generate ATP while inhibiting anabolic processes that consume ATP [120,122]. AMPK inhibits MTORC1 by phosphorylating TSC2 and RPTOR/raptor, leading to reduced anabolic processes and cell growth [123,124]. Moreover, the study has shown that AMPK can activate cofilin, an actin-binding protein that severs actin filaments, promoting actin cytoskeleton remodeling necessary for macropinocytosis [125]. By regulating actin dynamics and promoting cytoskeletal rearrangements, AMPK can enhance the cell’s ability to form macropinosomes and uptake extracellular fluid and nutrients. Under energy stress conditions, the activation of macropinocytosis by AMPK helps cells to scavenge extracellular nutrients, such as proteins, which can be degraded into amino acids and used to replenish intracellular nutrient pools. Knudsen et al. found that MTORC1 signaling can still be activated after muscle contraction even when AMPK activity is impaired. However, mice with deficient AMPK activity exhibit a reduced ability to stimulate protein synthesis following contraction. Moreover, increasing muscle glycogen content enhances MTORC1 signaling activation in AMPK-deficient mice and restores the protein synthesis response in these animals [126].

#### Oncogenic mutations—activated RAS

The *RAS* gene family includes *HRAS*, *KRAS*, and *NRAS* [127]. The proteins expressed corresponding to them regulate various signaling pathways involved in cell growth and survival. RAS proteins are small GTPases. RAS proteins function as molecular switches, cycling between an inactive GDP-bound state and an active GTP-bound state [128]. GTP binding activates RAS, enabling it to interact with downstream effectors. Common mutations, such as *KRAS* G12D [129], G12V [130,131], and G13D [131], prevent the hydrolysis of GTP, leading to persistent activation of *RAS*. Oncogenic *RAS* remains in its active GTP-bound state, continuously stimulating downstream signaling pathways. Activated RAS interacts with PI3K and activates it, which in turn activates the PI3K-AKT pathway [35]. Subsequent procedures were performed as described above. RAS activates the RAF-MAP2K/MEK-MAPK/ERK pathway, ultimately leading to the activation of MAPK/ERK [132–135] (Figure 3). MAPK phosphorylates and inhibits TSC2, reducing its GAP activity toward RHEB, a direct activator of MTORC1 [104,136]. This leads to increased levels of active RHEB-GTP, which directly activates MTORC1 at the lysosomal membrane. RAS activation can also promote the translocation of MTORC1 to the lysosomal surface, where it interacts with RHEB and other activators, facilitating its activation.

### Downstream effects of MTORC1 activation

#### Protein synthesis

**Figure 4.**
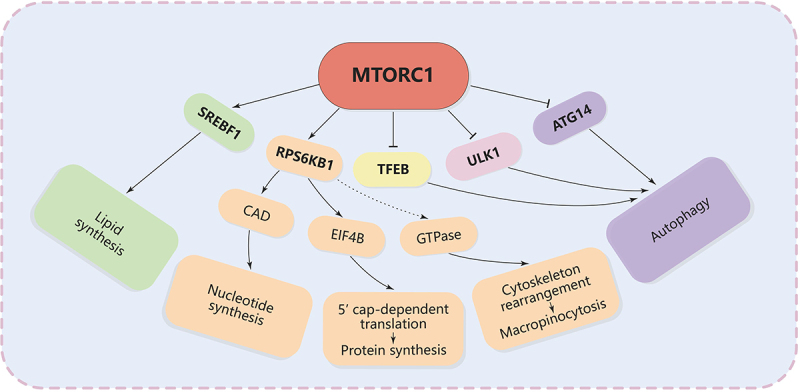
The major pathways downstream of MTORC1 signaling.

MTORC1 directly phosphorylates RPS6KB1/S6K1 (ribosomal protein S6 kinase B1) on its Thr389 residue, a crucial step for its activation [137]. Activated RPS6KB1 phosphorylates RPS6 (ribosomal protein S6) and other components involved in the translation machinery, enhancing the translation of mRNAs that encode ribosomal proteins and translation factors [138]. Phosphorylation of RPS6 increases the translation of mRNAs with a 5” terminal oligopyrimidine (TOP) tract, which encode components of the protein synthesis machinery such as ribosomal proteins and elongation factors [138,139]. RPS6KB1 also phosphorylates EIF4B (eukaryotic translation initiation factor 4B), enhancing its ability to stimulate the helicase activity of EIF4A, facilitating the unwinding of secondary structures in the 5” untranslated region/UTR of mRNAs and promoting the initiation of translation [140] (Figure 4). Böhm et al. reported that MTORC1 also phosphorylates EIF4EBP1 on multiple residues (e.g., Thr37/46, Ser65, Thr70), causing its dissociation from EIF4E [141]. Phosphorylation of EIF4EBP1 releases EIF4E, allowing it to bind to EIF4G and form the active EIF4F complex. The assembly of the EIF4F complex at the 5’ cap of mRNA promotes the recruitment of the 40S ribosomal subunit and other initiation factors, leading to the formation of the translation initiation complex and the start of protein synthesis [137].

#### Lipid synthesis

MTORC1 enhances the nuclear localization and activity of sterol regulatory element-binding transcription factors (SREBFs), leading to the upregulation of genes involved in lipid biosynthesis [142,143]. Peterson and colleagues demonstrated that MTORC1 regulates SREBF1 by controlling the nuclear entry of LPIN1 (lipin 1), a phosphatidic acid phosphatase [144]. The study found that inhibition of MTORC1 in the liver significantly impaired SREBF1 function and rendered mice resistant to high-fat, high-cholesterol diet-induced hepatic steatosis and hypercholesterolemia in a LPIN1-dependent manner [144]. However, the study still needs to delve deeper into how MTORC1 specifically regulates the multisite phosphorylation of LPIN1 and how this process is linked to the regulation of SREBF1 nuclear protein levels. Lipids are essential for the formation of cellular membranes, including the membranes of macropinosomes and other endocytic vesicles. Enhanced lipid biosynthesis supports the increased membrane trafficking associated with macropinocytosis. Interestingly, the group of Yi et al. reported a novel mechanism by which oncogenic activation of the MTOR signaling pathway suppresses ferroptosis in cancer cells via SREBF1-mediated lipogenesis, and demonstrated the potential therapeutic application of combining MTORC1 inhibition with ferroptosis induction in cancer treatment [145].

#### Nucleotide synthesis

MTORC1 enhances nucleotide biosynthesis by regulating key enzymes and pathways involved in the production of both pyrimidines and purines [100,105]. MTORC1 phosphorylates CAD (carbamoyl-phosphate synthetase 2, aspartate transcarbamylase, and dihydroorotase) through RPS6KB1. CAD is a multifunctional enzyme involved in pyrimidine synthesis, promoting the production of nucleotides. Increased pyrimidine synthesis supports cell proliferation by ensuring a sufficient supply of nucleotides for DNA replication and repair [146,147] (Figure 4). MTORC1 activation influences the synthesis of inosine monophosphate/IMP, a precursor for both adenine and guanine nucleotides. MTORC1 enhances the activity of PRPS (phosphoribosyl pyrophosphate synthetase), which generates phosphoribosyl pyrophosphate, a critical substrate for both purine and pyrimidine synthesis [105]. In addition, the study has shown that MTORC1 activation promotes the expression and activity of transcription factors such as MYC (MYC proto-oncogene, bHLH transcription factor) and HIF1A (hypoxia inducible factor 1 subunit alpha), which upregulate genes involved in nucleotide biosynthesis [148]. This series of activating effects increases purine production, ensuring an adequate supply of nucleotides required for DNA and RNA synthesis. Meanwhile, purine nucleotides also play crucial roles in a variety of cellular metabolic processes, including ATP production and signaling processes [102].

#### Inhibition of autophagy

Autophagy is a catabolic process that degrades and recycles cellular components, providing nutrients and maintaining cellular homeostasis [149–151]. The mechanistic target of MTORC1 is a key regulator that inhibits autophagy when nutrients and growth factors are abundant. The ULK1 complex is composed of ULK1 (unc-51 like autophagy activating kinase 1), ATG13 (autophagy related 13), ATG101 (autophagy related 101), and RB1CC1 (RB1 inducible coiled-coil 1) [152]. This complex is crucial for autophagy initiation. MTORC1 phosphorylates ULK1 directly at Ser757, which suppresses ULK1 activity and prevents the formation of an active ULK1 complex, thereby preventing the initiation of autophagy [153,154] (Figure 4). Kim et al. demonstrated that under nutrient-sufficient conditions, high MTOR1 activity prevents ULK1 activation by phosphorylating Ser757 and disrupting the interaction between ULK1 and AMPK [155]. Under glucose starvation conditions, AMPK directly activates ULK1 by phosphorylating Ser317 and Ser777, thereby promoting autophagy. Moreover, Park et al. found that under nutrient-deficient conditions the ULK1 complex mediates MTORC1 signaling to the autophagy initiation machinery via binding and phosphorylating ATG14 [156].

MTORC1 also mediates inhibition of the PIK3C3/VPS34 (phosphatidylinositol 3-kinase catalytic subunit type 3) complex and plays a significant role in controlling autophagy by affecting the nucleation phase of autophagosome formation [157]. The PIK3C3 complex typically comprises PIK3C3/VPS34, BECN1/VPS30/ATG6, PIK3R4/VPS15 (a serine/threonine kinase), NRBF2 and ATG14 [158]. This complex is pivotal for initiating autophagy through the production of phosphatidylinositol-3-phosphate (PtdIns3P) on early autophagic membranes. MTORC1 phosphorylates ATG14, inhibiting the kinase activity of PIK3C3 or altering the assembly and localization of the complex, thereby reducing PtdIns3P production and subsequent autophagosome formation [159].

TFEB (transcription factor EB) is a master regulator of lysosomal biogenesis and autophagy. It controls the expression of genes necessary for lysosome formation and the autophagy-lysosome pathway, critical for cellular clearance and recycling processes [160,161]. MTORC1 directly phosphorylates Ser211 of TFEB [162]. This phosphorylation promotes the binding of TFEB to members of the YWHA/14-3-3 phospho-serine binding proteins, preventing it from entering the nucleus. This sequestration inhibits TFEB’s ability to activate transcription of genes involved in lysosomal biogenesis and autophagy. Meanwhile, The activity of MTORC1, and consequently its effect on TFEB, is heavily influenced by cellular nutrient status [162]. Under nutrient-sufficient conditions, MTORC1 is active and maintains TFEB in a phosphorylated and inactive state. In contrast, nutrient deprivation also leads to reverse regulation of MTORC1 by TFEB. Nnah and colleagues reported how TFEB coordinates MTORC1 signaling and autophagy by promoting endocytosis, especially under nutrient-deprived conditions, revealing a novel mechanism for TFEB in activating MTORC1 [163].

While autophagy is generally a survival mechanism under stress conditions, its inhibition by MTORC1 ensures that cells prioritize growth over degradation when nutrients are abundant [164,165]. Cells dynamically regulate autophagy and MTORC1 activity in response to nutrient availability and stress, adapting their metabolism to environmental changes. This is particularly important for maintaining the anabolic state required for efficient macropinocytosis.

### Regulation of MTORC2 in nutrient metabolism

The regulation of MTORC2 in nutrient metabolism, while less prominent than MTORC1, plays a crucial supportive role in cellular homeostasis, particularly in response to environmental stresses and metabolic signals [100,137]. MTORC2 is a multi-protein complex that, like MTORC1, is sensitive to changes in nutrient availability, but it primarily regulates processes that affect cell survival, proliferation, and metabolic adaptation.

MTORC2 primarily functions as an effector of insulin-PI3K signaling [99,100,166] (Figure 3). When insulin binds to its receptor on the cell membrane, it activates PI3K, which catalyzes the conversion of PtdIns(4,5)P_2_ into PtdIns(3,4,5)P_3_. The MTORC2 complex, unlike MTORC1, is activated primarily by PI3K-generated PtdIns(3,4,5)P_3_ [100]. MSIN1, a component of MTORC2, plays a crucial role by binding to PtdIns(3,4,5)P_3_ through its PH domain (Figure 2). The MSIN1 PH domain inhibits MTORC2 catalytic activity in the absence of insulin, and this autoinhibition is relieved upon binding to PI3K-generated PtdIns(3,4,5)P_3_ at the plasma membrane [166]. There is a positive feedback loop between MTORC2 and AKT [167]. When MTORC2 is activated, it phosphorylates AKT at Ser473, which is necessary for AKT to become fully active. Fully activated AKT can then phosphorylate and further enhance the activity of MTORC2. This cycle amplifies the signaling through the PI3K-AKT pathway, contributing to cellular growth and survival [167]. Unexpectedly, MTORC2 signaling is also regulated by MTORC1, due to the presence of a negative feedback loop between MTORC1 and insulin-PI3K signaling [168]. MTORC1 phosphorylates and activates a protein called GRB10, a negative regulator of insulin and IGF1 receptor signaling [169,170]. GRB10 acts upstream of AKT and MTORC2, and its activation inhibits the insulin receptor signaling pathway by interfering with the insulin receptor and its associated proteins. Another important mechanism by which MTORC1 regulates MTORC2 is through the activation of RPS6KB1, which is a downstream effector of MTORC1. RPS6KB1 plays a critical role in regulating cell growth and protein synthesis [171]. It also participates in a negative feedback loop to suppress MTORC2 activation via IRS1 (insulin receptor substrate 1) [137].

**Figure 5.**
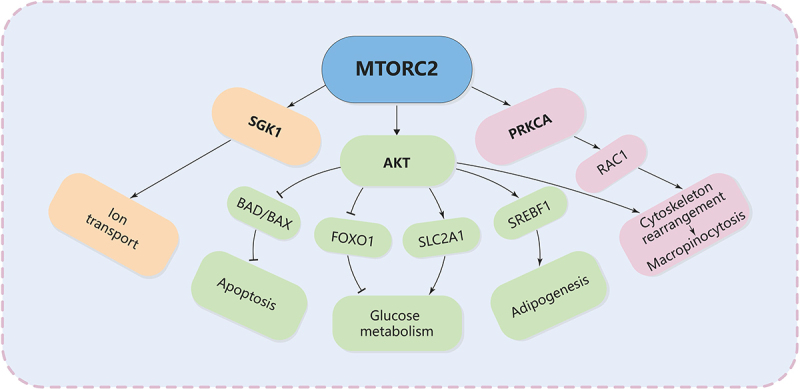
The major pathways downstream of MTORC2 signaling.

The most important downstream regulation of MTORC2 is the phosphorylation and activation of AKT, a key effector of insulin-PI3K signaling. This phosphorylation enables AKT to regulate several downstream targets involved in cell survival, apoptosis resistance, and metabolic homeostasis [168,172] (Figure 5). Activated AKT promotes cell survival by inhibiting pro-apoptotic factors such as BAD (BCL2 associated agonist of cell death) and BAX (BCL2 associated X, apoptosis regulator). MTORC2 also promotes the activity of anti-apoptotic proteins like BCL2 and MCL1, which help maintain mitochondrial integrity and prevent the initiation of cell death programs [173]. In nutrient-poor environments, where cellular stress is heightened, MTORC2 ensures that cells can continue to survive by enhancing these survival pathways. MTORC2 activation of AKT enhances glucose uptake by increasing the expression and translocation of SLC2A1/GLUT1 (solute carrier family 2 member 1) to the plasma membrane [174]. At the same time, once activated, AKT promotes cell survival, proliferation, and growth through the phosphorylation and inhibition of several key substrates, including the FOXO1 (forkhead box O1) transcription factors [175], the metabolic regulator GSK3B [176], and the MTORC1 inhibitor TSC2 [177]. AKT activation can upregulate SREBF1, which is involved in the synthesis of fatty acids and triglycerides, further supporting cellular growth and energy homeostasis. Study has shown that mice with liver-specific *rictor* knockout have a marked decrease in lipogenesis resulting from loss of SREBF1/SREBP-1c activity [178]. Finally, MTORC2 also phosphorylates and activates SGK1, another AGC-kinase that regulates ion transport as well as cell survival [179].

### The role of macropinocytosis-related proteins regulated by MTOR

Macropinocytosis allow cells to engulf extracellular fluid and solutes, enabling the internalization of vital nutrients, growth factors, and other essential molecules [17]. Macropinocytosis and MTOR signaling pathway are deeply intertwined, and their reciprocal regulation plays a pivotal role in cancer metabolism and tumor progression. The relationship between macropinocytosis and MTOR is bidirectional, with both pathways regulating each other in a feedback loop. When cancer cells internalize nutrients through macropinocytosis, the concentration of intracellular metabolites such as amino acids increases, which is then sensed by MTORC1, leading to the activation of cellular growth and metabolic processes [100,105]. In turn, MTORC1 regulates macropinocytosis by modulating the actin cytoskeleton, which is crucial for the formation of membrane ruffles and the engulfment of extracellular fluid [103,180].

Both RAC1 and CDC42, which are RHO-family GTPases, play essential roles in regulating actin polymerization and membrane protrusions during macropinocytosis [91,181]. The activation of RPS6KB1 by MTORC1 can indirectly regulate various scaffold proteins and kinases that interact with these GTPases, leading to the activation of RAC1 and CDC42 [182]. Activated RAC1 and CDC42 enhance the formation of filopodia and lamellipodia, structures critical for the initiation of macropinocytosis. While MTORC1 indirectly affects RAC1, MTORC2 also influences this small GTPase through a more direct pathway. PRKCA (protein kinase C alpha)/PKCα phosphorylation mediated by RICTOR in MTORC2 [183,184]. This can lead to an enhanced activation of RAC1 and promote macropinocytosis. The ACTR2-ACTR3 complex is a key actin-nucleating complex that is involved in the formation of branched actin filaments. Zhao et al. utilized a mouse model of inflammation-accelerated *Kras*^*G12D*^-driven early pancreatic carcinogenesis. They found that MTORC1 regulates the translation of RAC1 and the ACTR2-ACTR3 complex subunit ACTR3, whereas MTORC2 activates the ACTR2-ACTR3 complex by promoting AKT and RAC1 signaling [184].

All actin-binding proteins in Table 1 are regulated by MTORC1 and MTORC2. For example, MTORC1-RPS6KB1 can indirectly enhance the phosphorylation of LIMK, which in turn phosphorylate CFL (cofilin), rendering it inactive [57]. Moreover, both CFL and FMN (formin) can be activated by RHO family GTPases. AKT is a key component of the PI3K-AKT-MTOR signaling cascade and exerts regulatory control over profilin and capping proteins [51,55]. As a result, MTORC1 and MTORC2 indirectly regulate these proteins by affecting AKT activity (Table 1).

#### The possibility of developing new anti-cancer therapies through macropinocytosis

**Figure 6.**
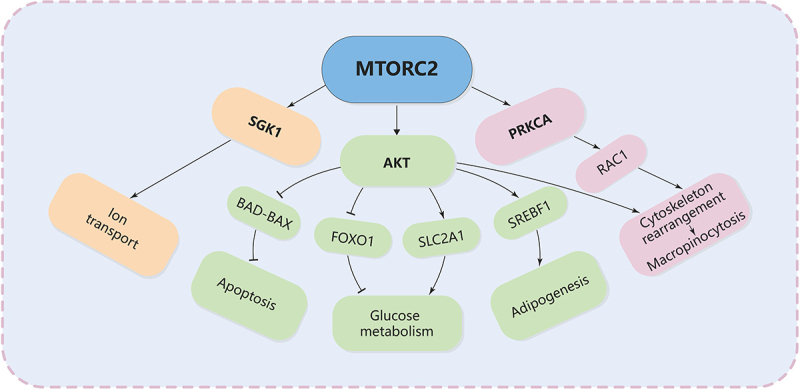
New anti-cancer therapies through macropinocytosis.

Traditional cancer treatments, while effective to a degree, often come with significant side effects and limitations due to their non-specificity. In recent years, there has been a growing interest in targeting cellular processes unique to cancer cells to develop more precise and effective treatments [84,185,186]. Due to the universality of macropinocytosis in some cancers, anti-cancer therapy targeting macropinocytosis has become an area of extensive research. According to the potential of macropinocytosis as an anti-cancer therapeutic target, it can be summarized in four aspects. Firstly, using the macropinocytosis mechanism, drugs can be delivered directly to the tumor cells, thus reducing the damage to the normal cells and improving the therapeutic effect. Secondly, inhibiting the macropinocytosis process can restrict the absorption of nutrients by cancer cells, thereby suppressing their proliferation and spread. Thirdly, specific molecular interventions can block the metabolism of energy and nutrients through the macropinocytosis pathway in cancer cells, thus limiting their survival and growth. Finally, excessive activation of macropinocytosis was promoted, resulting in specific cell death, which provided a new idea for the development of selective killing methods of tumor cells (Figure 6).

### Delivery of anti-cancer agents

Agents delivered by macropinocytosis can be broadly categorized into several types, each with unique properties and mechanisms of action. Delivering these agents via macropinocytosis can enhance their efficacy, reduce systemic toxicity, and overcome drug resistance, ensuring high intracellular drug concentrations while sparing normal cells. Here, we elaborated on the categories of agents delivered via macropinocytosis (Table 2). Meanwhile, polymeric nanoparticles, liposomes, and micelles encapsulating doxorubicin are designed to enhance its uptake via macropinocytosis. Enhanced intracellular delivery, reduced systemic toxicity, and improved efficacy in drug-resistant cancer cells. Nanoparticle formulations are engineered to optimize the encapsulation, protection, and controlled release of chemotherapeutic agents. There are several common methods used for incorporating drugs into nanoparticles: physical encapsulation [224,225], chemical conjugation [226], ionic or electrostatic interaction [227,228] and layer-by-layer assembly [229,230]. We also compared these several common nanoparticle drug encapsulation techniques (Table 3).

**Table 2.** Agents delivered by macropinocytosis.

Categories of agents	Agents	Novel synthesized compounds	Cells/Tumor	Ref
Chemotherapeutic Agents	Doxorubicin	Doxorubicin-loaded nanoparticles	U87 cells; U251 cells (glioblastoma)	[187]
		Doxorubicin-loaded nanoparticles	HeLa cells (*CD44* over expression cancer)	[188]
	Paclitaxel	ALB (albumin)-bound paclitaxel	Mouse RAW 264.7 cells (pancreatic cancer)	[189]
		ALB-bound paclitaxel	*KRAS*-mutant cancer cells (non-small cell lung cancer; pancreatic ductal adenocarcinoma)	[190]
	Cisplatin	Cisplatin chimeric nanoparticle	HCT-116 cells (colon cancer)	[191]
		Cisplatin by mesoporous silica nanoparticle	T47D cells; MCF-7 cells (breast cancer)	[192]
		Cisplatin by mesoporous silica nanoparticles	Huh7 cells (hepatocellular carcinoma)	[193]
Targeted Therapy Agents	Trastuzumab	Trastuzumab microcapsules	BT-474 cells (breast adenocarcinoma)	[194]
		Trastuzumab-modified nanoparticles	SK-BR-3 cells; MCF-7 cells (breast cancer)	[195]
	Imatinib	Imatinib nanocomposites	K562 cells (chronic myeloid leukemia)	[196]
	Erlotinib	Co-polymer nanocarrier for erlotinib	HeLa cells (cervical cancer)	[197]
		Erlotinib-loaded nanoparticles	A549 cells (non-small cell lung cancer)	[198]
Immunotherapeutic Agents	Checkpoint Inhibitors	Anti-PDCD1-loaded nanoparticles	MC38 cells (mouse colorectal cancer)	[199]
	Cytokines	RGD and IL13p polymeric nanoparticles	C6 cells (glioblastoma)	[200]
	Cancer Vaccines	CXCR4-targeted biomimetic nanovaccine	Dendritic cells	[201]
		Biomimetic nanovaccine (R837-αOVA-ApoE3-HNP)	Dendritic cells	[202]
	Oncolytic Viruses	Bio-reducible polymer complexed with oncolytic adenovirus	U343 cells; U87 cells (brain cancer)	[203]
Phototherapy Agents	Gold Nanoparticles	TAT-peptide-functionalized gold nanostars	BT549 cells (breast cancer)	[204]
		Polyethylene glycol-coated Ag@Au core-shell nanoparticles	U87 cells (glioblastoma)	[205]
		Polydopamine-coated gold-silver alloy nanoparticles	TPC-1 cells (papillary thyroid cancer)	[206]
Nucleic Acid-Based Therapies	Small Interfering RNA	Encapsulation of siRNA into cell-penetrating peptides lipid nanoparticles	HT1080 cells (fibrosarcoma)	[207]
		Encapsulation of siRNA into dioleylphosphate-diethylenetriamine conjugate lipid nanoparticles	HT1080 cells (fibrosarcoma)	[208]
		3“,3”’-bis-peptide-siRNA	A375 cells (melanoma)	[209]
		Encapsulation of siRNA into tris(2-aminoethyl)amine (TREN) and 3 linoleyl chain lipid nanoparticles	SK-HEP-1 cells (human liver cell line)	[210]
	MicroRNA	Encapsulation of *MIR34A* into stimulated lipoprotein-like nanoparticles	Glioma-initiating cells	[211]
		Chemically synthesized *MIR34A* and lipid-based delivery vehicles	A549 cells (non-small cell lung cancer)	[212]
		miRNA-encapsulated macrophage-targeting liposomes	RAW 264.7 cells	[213]
	mRNA vaccines	The poly-(β-amino ester) polymer mRNA core encapsulated within a lipid shell	B16 melanoma cells (melanoma)	[214]
		Endogenously LN-targeting lipid nanoparticles (113-O12B)	B16F10 melanoma cells (melanoma)	[215]
	Plasmid DNA	HIV-Tat-derived peptide/pDNA-Ca^2+^ nanoparticles	SKOV3 cells (ovarian cancer)	[216]
		The plasmid coding for TNF/TNFα (tumor necrosis factor) (pIRES2-EGFP-TNFα)	SW-1990 cells (pancreatic cancer)	[217]
		TNFSF10/TRAIL (TNF superfamily member 10)-expressing plasmid DNA	UMUC3 cells (bladder cancer)	[218]
	Antisense Oligonucleotides	Lipid-modified antisense Oligonucleotide targeting translationally controlled tumor protein	PC-3 cells (prostate cancer)	[219]
		RX-0047 lipid-albumin nanoparticles (RX-0047, an antisense oligonucleotide (ASO) against HIF1A (hypoxia inducible factor 1 subunit alpha)	KB cells (oral epidermoid cancer)	[220]
		Non-cationic transfection vector in the form of bottlebrush polymer-antisense oligonucleotide conjugates	NCI-H358 cells (non-small cell lung cancer with *KRAS* ^*G12C*^ mutation)	[221]
	CRISPR-Cas9	CRISPR-Cas9-ribonulceoproteins	RAW 264.7 cells	[222]
		Cas9-sgRNA-ribonucleoproteins	N2a eGFP/luc cells; CT26 eGFP/luc cells	[223]

**Table 3.** Comparison of several common nanoparticle drug encapsulation techniques.

Loading technique	Description	Advantages	Disadvantages
Physical encapsulation [224,225]	Drugs are encapsulated within the nanoparticle matrix without forming covalent bonds.	− Simple process − Suitable for a variety of drugs − Protects sensitive drugs from degradation	− Potential for premature drug release − Drug release heavily influenced by environmental conditions
Chemical conjugation [226]	Drugs are covalently bonded to the nanoparticle or its components, often via linkers that can be cleaved under specific conditions.	− Stable attachment − Controlled release triggered by environmental conditions - High drug-loading efficiency	− Complex synthesis − Potential toxicity of linkers − Requires careful control of reaction conditions
Ionic or electrostatic interaction [227,228]	Drugs are loaded onto nanoparticles through electrostatic attraction between oppositely charged molecules.	- High loading efficiency for charged molecules- No need for chemical modification of the drug	− Sensitive to ionic strength and pH changes − May lead to instability in biological environments
Layer-by-layer assembly [229,230]	Multilayered structures are formed on nanoparticles or other substrates through sequential adsorption of charged or complementary materials, allowing for the incorporation of different types of molecules.	− Allows for multiple drugs and functionalities − Precise control over release kinetics − Versatile layer composition	- Time-consuming process − Requires multiple steps and careful control of deposition conditions − Potential delamination

#### Chemotherapeutic agents

Doxorubicin intercalates DNA, inhibiting topoisomerase II and preventing DNA replication and transcription, leading to apoptosis [231,232]. Chen et al. demonstrated that doxorubicin-loaded polymeric nanoparticles can effectively cross the blood-brain barrier and achieve excellent cellular uptake through mechanisms such as macropinocytosis, allowing for drug accumulation in brain tumors and effectively killing tumor cells [187].

Paclitaxel stabilizes microtubules, preventing their disassembly and thereby inhibiting cell division [233]. Albumin-bound nanoparticles (Abraxane) exploit the natural tendency of albumin to be taken up by cells through macropinocytosis [190]. Improved solubility, targeted delivery to cancer cells with high macropinocytic activity, and enhanced antitumor activity. Cullis et al. reported a novel mechanism by which nab-paclitaxel activates macrophages through macropinocytosis and may influence the treatment of pancreatic cancer [189].

Cisplatin forms DNA adducts, leading to DNA crosslinking and apoptosis [234]. Cisplatin can covalently bind to a polymer to form a polymer-drug conjugate. This conjugation improves drug stability and drug delivery. Targeted delivery to tumor cells, reduced renal toxicity, and enhanced drug accumulation in cancer cells. The Palvai group reported the development of hyaluronic acid-layered chimeric nanoparticles (HA-CNPs) for targeted colon cancer therapy [191]. The study successfully encapsulated MAPK and PI3K pathway inhibitors along with the DNA-damaging drug cisplatin within the same nanoparticle, achieving active targeting of colon cancer through hyaluronic acid coating. Rapid internalization and localization of HA-CNPs in lysosomes led to effective inhibition of the MAPK-PI3K signaling hub, cell cycle arrest, and induction of apoptosis in colon cancer cells, demonstrating significant cytotoxicity.

#### Targeted therapy agents

Trastuzumab is a monoclonal antibody that targets the ERBB2 (erb-b2 receptor tyrosine kinase 2)/HER2 receptor, which is overexpressed in certain breast cancers [235]. By binding to ERBB2, trastuzumab inhibits cell proliferation and induces antibody-dependent cellular cytotoxicity. Trastuzumab can be conjugated to microcapsules to enhance targeting of ERBB2-positive cancer cells, improve drug delivery, and increase therapeutic efficacy. Liu et al. demonstrated that the conjugation of trastuzumab (TTZ) to microcapsules significantly improve their avidity and specificity for target cells, particularly for rod-shaped microcapsules [194].

Imatinib is a tyrosine kinase inhibitor that targets the BCR-ABL fusion protein, which is present in chronic myeloid leukemia/CML cells [236,237]. By inhibiting this kinase, imatinib blocks cell proliferation and induces apoptosis. Imatinib nanoparticles enhance their delivery via macropinocytosis, increasing intracellular drug concentration and efficacy. Increased intracellular concentration of imatinib in BCR-ABL-positive cells, reduced off-target effects, and improved efficacy [196].

Erlotinib is an EGFR tyrosine kinase inhibitor that blocks the activation of EGFR, inhibiting cell proliferation and inducing apoptosis in *EGFR*-mutant cancers [238]. The Bera group reported the development of a novel hypoxia-responsive pullulan-based co-polymer nanocarrier for erlotinib delivery to treat cervical cancer [197]. Cellular uptake studies indicated that these nanoparticles are internalized by HeLa cells through multiple endocytic pathways and effectively suppress cell proliferation and induce apoptosis.

#### Immunotherapeutic agents

Checkpoint inhibitors such as anti-PDCD1/PD-1 and anti-CTLA4 antibodies block inhibitory pathways in T cells, enhancing their activity against cancer cells [239,240]. Encapsulation of checkpoint inhibitors in nanoparticles can improve their delivery and uptake via macropinocytosis, ensuring higher local concentrations within the tumor microenvironment. Enhanced immune activation, improved targeting of the tumor site, and reduced systemic toxicity. Recent studies have shown that nanoparticles encapsulating anti-PDCD1 antibodies can be efficiently taken up by immune cells via macropinocytosis, leading to improved anti-tumor responses [199].

Cytokines such as interleukins and interferons modulate immune responses by activating immune cells and enhancing their anti-tumor activity [241,242]. Encapsulation of cytokines in nanoparticles protects them from degradation and enhances their delivery to immune cells via macropinocytosis. Prolonged circulation time, targeted delivery to the tumor microenvironment, and improved immune activation. Gao et al. developed a novel type of polymeric nanoparticles that carry the arginine-glycine-aspartate (RGD) peptide and IL13 (interleukin 13) peptide (IL13p) [200]. These nanoparticles have shown enhanced therapeutic efficacy and penetration by simultaneously targeting tumor cells and new blood vessels. At the same time, these nanoparticles altered cellular uptake mechanisms and changes in endocytosis pathways.

Cancer vaccines stimulate the immune system to recognize and attack specific tumor antigens, enhancing anti-tumor immunity [243–245]. Nanoparticles can encapsulate tumor antigens and adjuvants, facilitating their uptake by antigen-presenting cells/APCs via macropinocytosis and enhancing the immune response. Improved antigen presentation, enhanced activation of immune cells, and stronger anti-tumor immunity [243]. The group of Zhou developed an innovative biomimetic nanovaccine (R837-αOVA-ApoE3-HNP) that is efficiently internalized by dendritic cells via macropinocytosis, significantly enhancing the maturation and antigen presentation of dendritic cells (DCs) [202]. The study demonstrated the potential of this nanovaccine in augmenting T-cell immune responses and cancer immunotherapy, especially when used in combination with anti-PDCD1 antibodies.

Oncolytic viruses selectively infect and kill cancer cells while stimulating an anti-tumor immune response [246,247]. Encapsulation of oncolytic viruses in nanoparticles can enhance their delivery and uptake by cancer cells through macropinocytosis, enhance targeting to cancer cells, improve viral delivery, and increase immune activation [248,249]. Moon et al. reported a novel therapeutic approach using a pH-sensitive and bio-reducible polymer (PPCBA) complexed with oncolytic adenovirus (Ad) for cancer gene therapy [203]. The team demonstrated that the Ad-PPCBA complexes is able to target the low pH hypoxic tumor microenvironment and overcome the dependence on coxsackie and adenovirus receptor (CAR) expression. Moreover, the complex mediated internalization through macropinocytosis rather than the CAR-dependent endocytic pathway, preventing the adenovirus surface from innate immune responses.

#### Phototherapy agents

Photothermal therapies (PTT) have emerged as a promising modality in the treatment of various medical conditions, particularly cancer [250]. PTT leverages the conversion of light energy into heat. This localized heat generation can ablate tumor cells selectively, sparing surrounding healthy tissues [251–253]. The nanoparticles absorb the light energy and cause a rise in temperature that is lethal to cancer cells, leading to cell death via apoptosis or necrosis. Among the various agents utilized in PTT, gold nanoparticles/AuNPs are particularly noteworthy due to their unique optical properties, biocompatibility, and versatility in functionalization [254–256]. Yuan et al. reported on the enhanced intracellular delivery and efficient near-infrared photothermal therapy of TAT-peptide-functionalized gold nanostars [204]. Their study demonstrated that TAT-modified gold nanostars significantly outperform unmodified or PEGylated counterparts in cellular uptake, primarily through actin-driven lipid raft-mediated macropinocytosis. On BT549 breast cancer cells, photothermal therapy was achieved using an 850 nm pulsed laser at an irradiance of 0.2 W/cm^2^, which is below the maximum permissible exposure for skin.

#### Nucleic acid-based therapies

Small interfering RNA (siRNA) molecules can silence specific genes by promoting the degradation of target mRNA, effectively reducing the expression of oncogenes or other pathogenic genes in cancer cells [257]. siRNA can be encapsulated in lipid nanoparticles, polymeric nanoparticles, or other nanocarriers to enhance its stability and delivery via macropinocytosis [258–260]. The Sun group reported the optimization of the delivery pathway of 3“,3”’-bis-peptide-siRNA conjugates through nanocarrier architecture engineering [209]. They employed a combined nanochemistry strategy, demonstrating that “cicada pupa”-shaped MT-pp-siRNA/CLDs nanoparticles have enhanced siRNA protection and stability. These nanoparticles are internalized by melanoma cells through cave-mediated endocytosis and macropinocytosis (over 99.46%) and have reduced lysosomal degradation at a later stage. Eventually, the nanoparticle promoted the silencing of the mutant B-Raf protein mRNA (about 60% decrease).

MicroRNA (miRNA) are small non-coding RNAs that regulate gene expression by binding to target mRNA and inhibiting its translation or promoting its degradation [261]. Therapeutic miRNAs can restore the expression of tumor suppressor genes or inhibit oncogenes. miRNAs can be encapsulated in nanoparticles to protect them from degradation and enhance their delivery via macropinocytosis. Enhanced stability and delivery of miRNAs, improved gene regulation, and targeted effects on cancer cells [262]. Nanoparticles delivering miRNA-34a have been shown to be efficiently taken up by cancer cells via macropinocytosis, resulting in restored tumor suppressor activity and inhibited tumor growth [212].

Initially developed for the prevention of infectious diseases, mRNA vaccines have demonstrated a potent ability to activate cellular immunity. Consequently, delivering specific mRNA sequences to stimulate the immune system and elicit antigen-specific immune responses against tumors has emerged as an innovative approach to cancer treatment [263,264]. For example, Persano et al. developed a lipopolyplex (LPP) mRNA vaccine platform [214]. The LPP mRNA vaccine, which consists of a poly-(β-amino ester) polymer mRNA core encapsulated within a lipid shell, demonstrated superior efficacy in macropinocytosis, particularly in DCs. This is essential for initiating the immune response. Through a series of experiments, the LPP mRNA vaccine was shown to potently stimulate IFNB and IL12 expression, critical cytokines for DC maturation and anti-tumor immunity. In a B16-OVA melanoma lung metastasis model, the LPP mRNA vaccine led to a significant reduction in tumor nodules, highlighting its potent anti-tumor activity [214].

Plasmid DNA vectors are circular DNA molecules that can be customized to carry therapeutic genes, including tumor suppressors, suicide genes, or cytokines [265]. To protect the plasmid DNA from degradation in the extracellular environment and enhance its delivery, it is encapsulated within nanoparticles. These nanoparticles are often made from materials like cationic lipids or polymers that can compact the DNA and facilitate cellular uptake [266]. Cancer cells can internalize the DNA-loaded nanoparticles through macropinocytosis. Once inside the nucleus, the plasmid’s promoter drives the transcription of the therapeutic gene. Proteins produced can directly cause the death of cancer cells. Su and colleagues demonstrated that the addition of Ca^2+^ effectively condensed Tat-pDNA complexes into smaller, nontoxic nanoparticles, which are internalized by ovarian cancer cells primarily through macropinocytosis [216]. Meanwhile, researchers discovered that the overexpression of *HSPA9/GRP75* notably enhanced macropinocytosis in ovarian cancer cells, thereby synergizing with Tat-pDNA-Ca^2+^ nanoparticles to significantly amplify the cytotoxic effects on tumor cells.

Antisense oligonucleotides (ASOs) are short, single-stranded DNA or RNA molecules that bind to complementary mRNA sequences, blocking their translation or promoting their degradation, thus reducing the expression of target genes [267,268]. ASOs can be delivered using nanoparticles to protect them from degradation and enhance their uptake via macropinocytosis [269]. It can enhance stability and delivery of ASOs, improved gene silencing efficiency, and targeted effects on cancer cells. Karaki et al. developed a novel lipid-modified ASO targeting translationally controlled tumor protein (TCTP-LASO) [219]. They found that TCTP-LASOs, compared to unmodified ASOs, exhibit faster cellular penetration and higher efficiency in inhibiting TCTP expression, are internalized by macropinocytosis, and delay tumor progression in vivo with lower toxicity.

The CRISPR-Cas9 system enables precise gene editing by introducing double-strand breaks at specific genomic locations, allowing for the disruption of oncogenes or the correction of genetic mutations in cancer cells [270,271]. Nanoparticles encapsulated in the CRISPR-Cas9 components (Cas9 protein and guide RNA) can be delivered via macropinocytosis [272]. This was able to improve the stability and delivery of the CRISPR-Cas9 components, targeted gene editing, and potentially permanent genetic modification. Nanoparticles delivering CRISPR-Cas9 components have been shown to be efficiently taken up by cancer cells through macropinocytosis, resulting in effective gene editing and tumor suppression [222].

### Inhibition of macropinocytosis as an anti-cancer therapy

Macropinocytosis shows strong activity in many cancer cells. This enhanced activity allows cancer cells to uptake both extracellular proteins and other nutrients. By inhibiting macropinocytosis, it is possible to starve cancer cells of these essential resources, thereby inhibiting tumor growth and inducing cell death. Here, we elaborate on drugs that inhibit macropinocytosis.

#### Agents that inhibit intracellular pH regulation

Amiloride and its derivatives inhibit the Na^+^/H^+^ exchanger, which is involved in the regulation of intracellular pH and macropinosome formation. These compounds prevent the acidification required for the maturation and function of macropinosomes, thereby inhibiting the uptake of extracellular nutrients and reducing cancer cell survival [273]. Yao et al. investigated how the upregulation of SLC9A7/NHE7 (solute carrier family 9 member A7) in hepatocellular carcinoma (HCC) enhances the ability of tumor cells to take up small extracellular vesicles (sEVs) through macropinocytosis. By inhibiting macropinocytosis with 5-(N-ethyl-N-isopropyl)-amiloride (EIPA), the entry of sEVs can be restricted, thereby reducing the cells’ invasiveness [274].

Recent studies have begun to explore the synergistic potential of combining macropinocytosis inhibitors with other therapeutic strategies to enhance the efficacy of cancer treatment. One such approach involves the use of ubiquitin-proteasome system (UPS) inhibitors. The UPS plays a critical role in regulating cellular protein homeostasis, and its dysregulation is often observed in cancer cells, contributing to their survival and proliferation [275,276]. By targeting the UPS, inhibitors can disrupt the protein degradation machinery of cancer cells, leading to the accumulation of key regulatory proteins and subsequent cell death. Wang et al. reported the combination treatment of EIPA and bortezomib/BTZ resulted in a notable decrease in cell viability, with synergy scores calculated by SynergyFinder indicating a highly synergistic effect [277]. Apoptosis analysis showed that the dual-drug strategy induces higher rates of early and late apoptosis compared to single treatments. Tu et al. also found that by inhibiting endocytic pathways, especially clathrin – dependent and caveolin-dependent endocytosis as well as macropinocytosis, the communication between multiple myeloma (MM) cells mediated by sEVs can be reduced, thereby enhancing the anti-MM properties of bortezomib [278].

#### Agents that disrupt the actin cytoskeleton

Cytochalasin D is a fungal toxin. Cytochalasin D binds to the barbed ends of actin filaments, preventing their polymerization and causing depolymerization of existing filaments [279]. Cytochalasin D prevents the formation of membrane ruffles and macropinosomes, effectively blocking macropinocytosis. The study found that cytochalasin D inhibited macropinocytosis in HeLa cells, which led to a reduction in the transduction efficiency of recombinant adeno-associated virus/rAAV [280].

Ruthenium-based compounds, particularly ruthenium nanoparticles, interfere with cellular redox balance and disrupt the actin cytoskeleton [281]. Ruthenium compounds generate reactive oxygen species/ROS that induce oxidative stress, leading to cytoskeletal reorganization and inhibition of macropinosome formation, thereby impairing nutrient uptake and promoting cell death [282].

### Inhibition of degradation and recycling in macropinosomes as anti-cancer therapy

After macropinosomes are formed, the internalized substances such as proteins, sugars, and other nutrients need to be processed and utilized by the cancer cells. This involves degradation in lysosomes and subsequent metabolism to generate energy and biosynthetic precursors. Drugs that interfere with these processes can effectively incapacitate macropinocytosis and starve cancer cells of essential nutrients.

#### Lysosomal inhibitors

Lysosomal inhibitors are broadly divided into two categories. One is increasing lysosomal pH to interfere with its function, and the other is protease inhibitors that block the degradation of proteins within lysosomes.

Chloroquine (CQ) and hydroxychloroquine/HCQ are antimalarial drugs and are also lysosomotropic agents [283]. These drugs accumulate in lysosomes and increase their pH, disrupting the activity of the lysosomal enzymes. These enzymes are required for the degradation of macropinosome contents [284]. This impairs the degradation of macropinosome contents and the recycling of nutrients, resulting in reduced nutrient availability to cancer cells. However, the Mauthe group showed that CQ mainly inhibits autophagy by impairing autophagosome fusion with lysosomes rather than by affecting the acidity and/or degradative activity of this organelle [285]. Lys05 is a more potent analog of chloroquine that also inhibits lysosomal acidification and function, preventing the degradation of macropinosome contents [286]. Lys05 induces lysosomal dysfunction, leading to the accumulation of autophagic and macropinocytic vesicles, reducing nutrient availability, and sensitizing cancer cells to nutrient deprivation. Bafilomycin A_1_ is a specific inhibitor of the V-ATPase in lysosomes, preventing lysosomal acidification [284,287]. This disrupts the breakdown of macropinosome contents and prevents nutrient recycling.

#### Autophagy inhibitors

Autophagy and macropinocytosis are closely linked processes within cells. While macropinocytosis involves the internalization of extracellular fluid and nutrients, autophagy is the process through which cells degrade and recycle intracellular components, including damaged organelles and proteins [17,47,105,288]. Autophagy also plays a crucial role in the processing and recycling of macropinosome contents after they are delivered to lysosomes [149,150,165]. By inhibiting autophagy, it is possible to impair the degradation and recycling of macropinosome contents, depriving cancer cells of essential nutrients and promoting cell death. Since both pathways ultimately converge on lysosomes for the degradation and processing of engulfed materials, any disruption in lysosomal function may impair the efficiency of macropinosome processing, thereby affecting the cell’s ability to effectively utilize internalized nutrients. Consequently, any autophagy inhibitors that affect lysosomal function or degradation processes may also affect macropinocytosis.

3-Methyladenine is a class I PI3K and class III phosphatidylinositol 3-kinase (PtdIns3K) inhibitor that blocks the nucleation of autophagosomes [289]. By inhibiting this step, 3-MA prevents the formation of autophagosomes, thereby impairing autophagy and macropinocytosis. Wu et al. showed that 3-MA inhibits autophagy by blocking class III PtdIns3K, leading to reduced autophagosome formation and impaired degradation of macropinocytic vesicles, resulting in nutrient deprivation [290]. Spautin-1 inhibits USP10 and USP13, two deubiquitinating enzymes that regulate the stability of BECN1, a key autophagy protein [291]. By promoting the degradation of BECN1, Spautin-1 inhibits autophagy at the initiation stage, impairing the processing of macropinocytic vesicles. Liu et al. found that Spautin-1 promotes the degradation of BECN1, leading to inhibition of autophagy and accumulation of undegraded macropinosomes, resulting in reduced nutrient recycling and impaired cancer cell growth [292].

Recent studies have shown that the inhibition of autophagy can, under certain conditions, lead to the activation of macropinocytosis. This compensatory activation is primarily observed in cancer cells, which often exhibit an increased demand for nutrients to support rapid growth and proliferation. When autophagy is inhibited, these cells may experience metabolic stress due to the accumulation of cellular waste and a shortage of molecular building blocks normally recycled by autophagy. To compensate for this deficit, cancer cells can enhance macropinocytosis as an alternative nutrient acquisition strategy. Su et al. demonstrated that when autophagy is suppressed, cancer cells can enhance macropinocytosis to acquire essential nutrients, with NFE2L2/NRF2 playing a central regulatory role in this process [293]. Therefore, autophagy inhibitors also promote macropinocytosis. Furthermore, the research discovered that simultaneous blockade of autophagy and macropinocytosis significantly inhibits tumor growth, offering new strategies for cancer therapy.

Autophagy inhibitors may promote macropinocytosis by activating it as a compensatory mechanism, but at the same time, if they affect lysosomal function, they may also inhibit the efficiency of macropinocytosis. The dual outcomes of autophagy inhibition on macropinocytosis underscore the need for a strategic approach when considering therapeutic interventions targeting these pathways in cancer. If autophagy inhibitors inadvertently activate macropinocytosis, this could potentially counteract the intended therapeutic effects by allowing cancer cells to continue acquiring nutrients through alternative pathways. This scenario highlights the importance of a comprehensive understanding of the metabolic state and adaptive capabilities of cancer cells when designing therapeutic strategies. By simultaneously blocking both pathways, it may be possible to more effectively starve cancer cells of necessary nutrients, thus enhancing the therapeutic outcome.

### Methuosis as an anti-cancer therapy

Methuosis occurs when macropinocytosis is hyperstimulated, leading to the formation of numerous large vacuoles in the cytoplasm [82,83]. Under normal conditions, macropinosomes fuse with lysosomes, where their contents are degraded and recycled. However, when macropinocytosis is excessively activated, these vesicles accumulate in the cytoplasm and continue to grow as more extracellular fluid becomes internalized [24,84]. The macropinosomes either fail to fuse with lysosomes or, when fusion occurs, the lysosomes are unable to efficiently degrade the contents. This leads to the persistence of large, fluid-filled vacuoles in the cytoplasm. The continuous expansion of these vacuoles eventually compromises the cell membrane’s integrity. Additionally, the metabolic burden of maintaining such an excessive macropinocytic activity leads to an energy crisis within the cell, further pushing it toward cell death [83,84]. Therefore, it is critical to identify and develop novel compounds that can effectively hyperstimulate macropinocytosis.

Since methuosis is often linked to oncogenic *RAS* activity, cancers with *RAS* mutations are particularly vulnerable to therapies that induce this form of cell death [33,294]. RAS activators drive the macropinocytosis pathway beyond its limit, and methuosis can selectively target these cancer cells. The Fang group extracted the compound spiropachysine A from the traditional Chinese medicine *Pachysandra axillaris* Franch. var. styiosa (Dunn) M. Cheng, which has an antiproliferative effect on hepatocellular carcinoma [295]. Interestingly, the study found that spiropachysine A induced methuosis is associated with the activation of RAS-RAC1 signaling pathway, but it does not depend on the known methuosis-related targets MTORC1/MTORC2 or CSNK2 (casein kinase 2). Increasing reports demonstrate that many small molecule compounds could specifically induce methuosis in tumor cells while showing little or no effect on normal cells [296]. Bello et al. found that a kind of trehalose, which is found in various dietary sources and used as a safe nutrient supplement [297]. They noted a pleiotropic effect on the tumor cells. The study reported the effects of trehalose on macropinocytosis in *NF1*-deficient glioblastoma cells and found its ability to induce cell death through methuosis. The Wu group designed and synthesized a series of derivatives. Notably, derivative 12A demonstrated the ability to induce methuosis selectively in cancer cells without affecting normal human cells [298]. The study elucidated that 12A-induced vacuoles originate from macropinosomes and are associated with endoplasmic reticulum stress and the activation of the MAPK/JNK signaling pathway. The compound also significantly inhibited tumor growth in an MDA-MB-231 ×enograft mouse model, showcasing its potential as a lead compound for the development of methuosis inducers. However, the report’s limitation is the need for further exploration of the precise molecular targets and mechanisms underlying the anti-cancer activity of 12A. Our comprehension toward the role of methuosis in cancers is still limited and the detailed mechanisms of how different small molecule methuosis inducers affect cancer cells have not been fully understood.

The exploration of macropinocytosis as a therapeutic target presents promising avenues for the development of novel anti-cancer therapies. By leveraging the unique properties of macropinocytosis, researchers are investigating multiple strategies to exploit this pathway in cancer cells. Collectively, these approaches underscore the potential of targeting macropinocytosis to create innovative and effective cancer therapies.

## Conclusion and perspectives

This review provides a comprehensive exploration of the therapeutic potential of targeting macropinocytosis, a nonselective form of endocytosis, for the development of novel anti-cancer therapies. Macropinocytosis is particularly active in certain cancer cells, allowing them to uptake large amounts of extracellular nutrients necessary for their rapid growth and survival [13,14,20]. There are currently four main treatment strategies: enhancing drug delivery via macropinocytosis, inhibiting macropinocytosis to starve cancer cells, blocking the degradation and recycling of macropinosome contents to impair cancer cell survival, and inducing methuosis – a form of cell death driven by excessive macropinocytosis. The proposal of these strategies has not only enriched our understanding of the metabolic dependence of cancer cells, but also provided innovative ideas for the development of new anti-cancer therapies. Macropinocytosis presents a novel target for anti-cancer therapy, with multiple clinically used products and novel entities under clinical trials exploring its potential. Abraxane is the most classic example, which has been used in breast cancer, pancreatic cancer and non-small cell lung cancer [190]. The most extensively studied aptamer, AS1411, has been tested in phase II clinical trials and is internalized via macropinocytosis in DU145 prostate cancer cells, glioma cells, and liver cells overexpressing nucleolin [299].

However, we still face the challenge of translating these research findings into clinical applications. One of the major challenges is achieving selectivity in targeting macropinocytosis in cancer cells without affecting normal cells that also utilize this process, such as immune cells. Developing drugs that can discriminate between cancerous and non-cancerous cells remains a significant hurdle. Ensuring that drugs delivered via macropinocytosis remain stable, reach their target cells in adequate concentrations, and are not prematurely degraded or expelled by the cells is another challenge that needs to be addressed to maximize therapeutic efficacy. While inducing methuosis presents a novel therapeutic approach, the precise molecular mechanisms and regulatory pathways underlying this form of cell death are not fully understood. Further research is needed to elucidate these mechanisms and optimize methuosis-inducing therapies.

Overall, targeting macropinocytosis represents a novel and innovative approach that could significantly advance the treatment of cancers that rely on this pathway for survival. Through continuous research and innovation, we look forward to developing more effective and safer anti-cancer therapies that will bring new hope to patients.
